# A Novel Assay Reveals a Maturation Process during Ascospore Wall Formation

**DOI:** 10.3390/jof3040054

**Published:** 2017-10-02

**Authors:** Kai Zhang, Leor Needleman, Sai Zhou, Aaron M. Neiman

**Affiliations:** Department of Biochemistry and Cell Biology, Stony Brook University, Stony Brook, NY 11794-5215, USA; zkb422@hotmail.com (K.Z.); leorneedleman@gmail.com (L.N.); zs1001365@gmail.com (S.Z.)

**Keywords:** ascospore, spore wall, dityrosine, chitosan, tyrosine, sporulation

## Abstract

The ascospore wall of the budding yeast *Saccharomyces cerevisiae* consists of inner layers of similar composition to the vegetative cell wall and outer layers made of spore-specific components that confer increased stress resistance on the spore. The primary constituents of the outer spore wall are chitosan, dityrosine, and a third component termed Chi that has been identified by spectrometry but whose chemical structure is not known. The lipophilic dye monodansylpentane readily stains lipid droplets inside of newly formed ascospores but, over the course of several days, the spores become impermeable to the dye. The generation of this permeability barrier requires the chitosan layer, but not dityrosine layer, of the spore wall. Screening of a set of mutants with different outer spore wall defects reveals that impermeability to the dye requires not just the presence of chitosan, but another factor as well, possibly Chi, and suggests that the *OSW2* gene product is required for synthesis of this factor. Testing of mutants that block synthesis of specific aromatic amino acids indicates that *de novo* synthesis of tyrosine contributes not only to formation of the dityrosine layer but to impermeability of the wall as well, suggesting a second role for aromatic amino acids in spore wall synthesis.

## 1. Introduction

The formation of ascospores is a defining feature of ascomycete fungi [1]. Ascospores are formed after meiosis and are surrounded by a specialized cell wall, hereafter referred to as the spore wall, which is distinct in both composition and morphology from the cell wall of vegetative cells. The best characterized ascospore wall is that of the baker’s yeast *Saccharomyces cerevisiae* [2].

In response to nitrogen starvation in the presence of a poor carbon source, diploids of *S. cerevisiae* exit the mitotic cell cycle, undergo meiosis to generate four haploid nuclei and envelope each of those nuclei inside of newly formed plasma membranes to create four spores [2]. Each spore is surrounded by a spore wall and all four spores are contained within the remnant of the mother cell, the ascus. The spore wall is a more extensive structure than the vegetative cell wall and consists of inner layers of mannoproteins and β-glucans, which are similar in composition to the vegetative cell wall, and outer layers consisting of spore-specific components [3,4,5]. The primary component of the outer spore wall is the β-1,4-glucosamine polymer, chitosan [3]. This is synthesized through the action of the sporulation-specific deacetylases Cda1 and Cda2 on chitin produced by the chitin synthase Chs3 [6,7,8]. The chitosan is assembled into a visible layer outside of the mannan and β-glucan and then a layer consisting primarily of the cross-linked di-amino acid dityrosine is formed outside of the chitosan [9,10]. Chitosan is essential for assembly of the outer spore wall, so mutants in *CHS3* lack both the chitosan and dityrosine layers [8].

Dityrosine is synthesized in the spore cytoplasm by the serial action of two enzymes, Dit1 and Dit2 and then exported to the spore wall through a transporter, Dtr1, in the spore plasma membrane [11,12]. Once exported, the dityrosine is assembled into a large polymer on the surface of the spore wall, although the structure of the polymer built from the dityrosine has not been determined [9]. The dityrosine polymer is a polyaromatic compound and may serve an analogous role to the melanin found in some fungal cell walls [13]. Additionally, nuclear magnetic resonance (NMR) studies of isolated outer spore walls reveal the presence of another component, distinct from both chitosan and dityrosine, termed Chi [14]. The chemical nature of Chi is unclear, though its presence is independent of dityrosine and it may serve to help connect the dityrosine and chitosan layers [14].

These unique outer spore wall components are the distinguishing feature of ascopores and confer on the spore increased resistance to a wide variety of environmental insults including high salt, temperature, low and high pH, as well as digestion in the gut of insects [15]. Spores are also more resistant to exposure to organic solvents such as diethyl ether, and ether exposure is commonly used to test for spore wall integrity [16]. How the spore wall confers resistance to such a variety of insults is not clear. It is likely that different components of the spore wall contribute resistance to different agents. For example, the dityrosine layer creates a barrier to the diffusion of proteins in and out of the spore wall and so *dit1*∆ mutants are sensitive to treatment with glucanase enzymes; however, *dit1*∆ mutants do not display strong sensitivity to ether vapor [14,17].

In addition to the genes encoding enzymes responsible for chitosan and dityrosine synthesis, several additional genes important for outer spore wall assembly have been identified [12,14,17,18]. Mutations in some of these genes lead to reduced levels of Chi and dityrosine in the spore wall, some show only reduced dityrosine, while others display increased sensitivity to environmental challenge but lack obvious spore wall defects [14,17,18]. In most cases, it is not clear how the gene products involved promote spore wall assembly.

Staining of spores with the hydrophobic dye monodansylpentane (MDH) revealed that wild-type spores become impermeable to the dye over the course of a few days after spore formation. This maturation requires the chitosan layer of the outer spore wall, but not the dityrosine layer. A set of previously identified spore wall mutants was assayed for MDH permeability. The results of these tests distinguish between mutants that specifically affect dityrosine synthesis and assembly versus those with broader effects on the outer spore wall. Mutants defective in aromatic amino acid biosynthesis were also examined for effects on spore wall formation. The results reveal that *de novo* tyrosine synthesis contributes to the formation of dityrosine layer precursors. In addition, tyrosine is important for spore wall maturation independently of its role as a dityrosine precursor, suggesting that derivatives of tyrosine contribute to other aspects of spore wall structure.

## 2. Materials and Methods

### 2.1. Yeast Strains and Plasmids

Standard media were used unless otherwise noted [19]. The strains used in this study are listed in Table 1. The integrating plasmid pRS304 [20] was used to introduce *TRP1* into strains in the AN120 background. The pRS304-PET10-RFP plasmid was constructed by PCR amplification of a *PET10-GFP* fusion from the GFP-tagged yeast collection strain [21] from −700bp upstream of the *PET10* start codon to the 3′ end of the *CYC1* terminator. This PCR fragment was digested with BglII and ClaI sites, for which sites were introduced at the 5′ and 3′ ends respectively, and cloned into BamHI/ClaI cut pRS304. This plasmid was then cut with PacI and AscI to release the GFP coding region and the vector backbone ligated with a PCR fragment carrying the RFP coding region and PacI and AscI digested ends. Haploids AN117-4B and AN117-16D were transformed with EcoRI digested pRS304-PET10-RFP to target integration of the plasmid to the *PET10* promoter region. The resulting haploids, yKZ72 and yKZ70, respectively, were mated to generate yKZ104. Homozygous deletions of *DIT2* and *OSW2* were generated by PCR-mediated replacement with the kanamycin and hygromycin resistance markers, respectively [22,23], in both AN117-4B and AN117-16D and mating of the resulting haploids to generate ySZ233 and ySZ265. The diploids yKZ108 and yKZ110 were generated by deleting *PHA2* and *TYR1*, respectively, with the kanamycin marker in the same manner, with the additional step that the resulting diploids were then transformed with Bsu36I digested pRS304. The diploids yKZ107, yKZ111 and yKZ112 were made by transformation of AN120, AN262, and AN264, respectively, with Bsu36I digested pRS304.

### 2.2. MDH Staining Assay

Sporulation was performed by replica plating of patches grown on yeast extract peptone dextrose (YPD) medium to SPO plates (2% agar, 1% KOAc, 0.05% yeast extract, 0.05% amino acid mixture without arginine, 0.05% glucose) and incubation of the plates at 23 °C. For staining of cells with MDH, cells were scraped off the plate with a toothpick and resuspended in 100 microliters of 2% KOAc. One microliter of a 5 mM solution of monodansylpentane (Abgent, San Diego, CA, USA) dissolved in DMSO was added to the suspension and the cells were incubated for 5 min at 23 °C. Cells were pelleted, washed twice with 200 microliters of 2% KOAc, resuspended in 20 microliters of 2% KOAc and then 1.5 microliters transferred to microscopy slides for inspection.

### 2.3. Dityrosine Fluorescence Assay

Cells were sporulated in liquid culture. Overnight cultures were grown to saturation at 30 °C in YPD medium and then diluted into yeast extract peptone acetate (YPA) medium for 12 h. The OD_660_ of these cells were measured, the cells pelleted, washed once in 2% KOAc and then resuspended in KOAc at an OD_660_ of 1.2. Cells were incubated in 2% KOAc for 48 h and then spores examined by fluorescence microscopy. The dityrosine fluorescence intensity was calculated for each spore by taking the average intensity at two points in the spore wall and subtracting the background fluorescence outside of the spore.

### 2.4. Microscopy

For the lipid droplet assay, stained cells were examined on a Zeiss Axioplan 2 microscope (Zeiss, Thornwood, NY, USA) using a blue fluorescence filter set (ex. 360 nm, em. 460 nm) for MDH signal and a red fluorescence set (ex. 540 nm, em. 605 nm) for the Pet10-RFP signal and DIC optics for transmitted light. Images were obtained using Zeiss Axiovision software. For the dityrosine assay, images were collected on a Zeiss Observer Z1 microscope using a dityrosine-optimized filter set (ex. 330 nm, em. 410 nm) and a fixed 2 s exposure time for all images. Fluorescence intensity was quantified using Zeiss Zen software.

### 2.5. Ether Tests

Strains were sporulated in liquid as for the dityrosine assay. After 48 h in 2% KOAc, five-fold serial dilutions of sporulated cells were spotted on two YPD plates in glass petri dishes. One plate served as a plating control and the other was inverted for 30 min over filter paper (Whatman, Maidstone, United Kingdom) that had been soaked with 6 mL of di-ethyl ether (J.T. Baker, Phillipsburg, NJ, USA). Both plates were incubated at 30 °C for two days before photographing.

## 3. Results

### 3.1. Staining with Monodansylpentane Reveals a Maturation Process during Spore Wall Development

**Figure 1:**
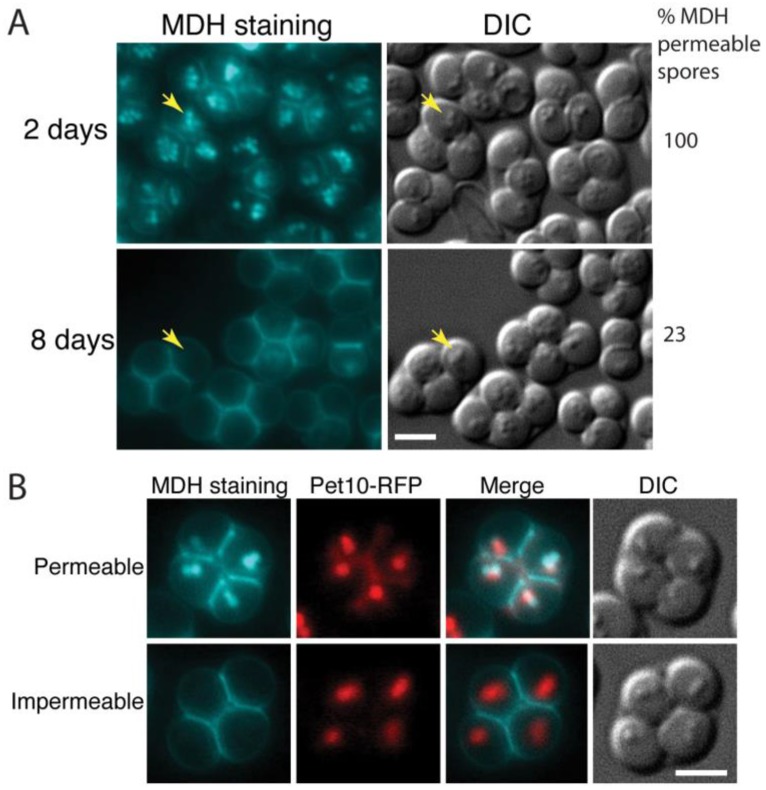
Older spores become impermeable to MDH. (**A**) Asci formed by the wild-type (AN120) strain after incubation on SPO plates for two days or eight days were stained with MDH and observed by fluorescence (blue staining) and light microscopy. The percentage of spores in the culture that displayed internal MDH staining is given at right (at least 500 spores counted). Yellow arrows indicate examples of lipid droplets visible by differential interference contrast (DIC) microscopy. Scale bar = 3 microns. (**B**) Five-day old asci of a wild-type strain expressing the lipid droplet marker Pet10-RFP (yKZ104) were stained with MDH and examined by fluorescence microscopy. Examples of asci containing MDH permeable or impermeable spores are shown. Scale bar = 3 microns.

The lipophilic dye monodansylpentane (MDH) can be used to specifically label lipid droplets in yeast [27]. Earlier work revealed that lipid droplets separate into two populations during sporulation with some segregating inside the forming spore and others remaining outside the spore and associating with the spore wall as it forms [5,14,28]. This latter population of droplets disappears during spore wall assembly so that in newly formed asci, bright MDH staining is predominantly seen inside of the spores ([28]; Figure 1A). By contrast, when 8-day old asci were similarly stained with MDH, the dye was mainly visible in the ascal space between the spores and the majority of spores failed to show internal staining or showed only faint internal staining (Figure 1A).

This loss of internal MDH staining might indicate the consumption of neutral lipids and disappearance of droplets within the spores. However, apparent lipid droplets can still be seen as small ‘bumps’ in differential interference contrast (DIC) optics in the spores from both time points (Figure 1A). To confirm that lipid droplets are still present in the older spores, asci of a strain expressing a fluorescently tagged lipid droplet protein, Pet10-RFP, were examined by MDH staining five days after transfer to SPO medium (Figure 1B). In asci with spores that were permeable to the dye, the Pet10-RFP and MDH staining colocalized, as expected. In spores that were impermeable to MDH, the localization of Pet10-RFP into discrete foci coincident with the lipid droplets seen in DIC was unchanged. This suggests that the lack of MDH staining in older spores is due to changes in permeability of the spores over time and not disappearance of the lipid droplets within spores.

**Figure 2:**
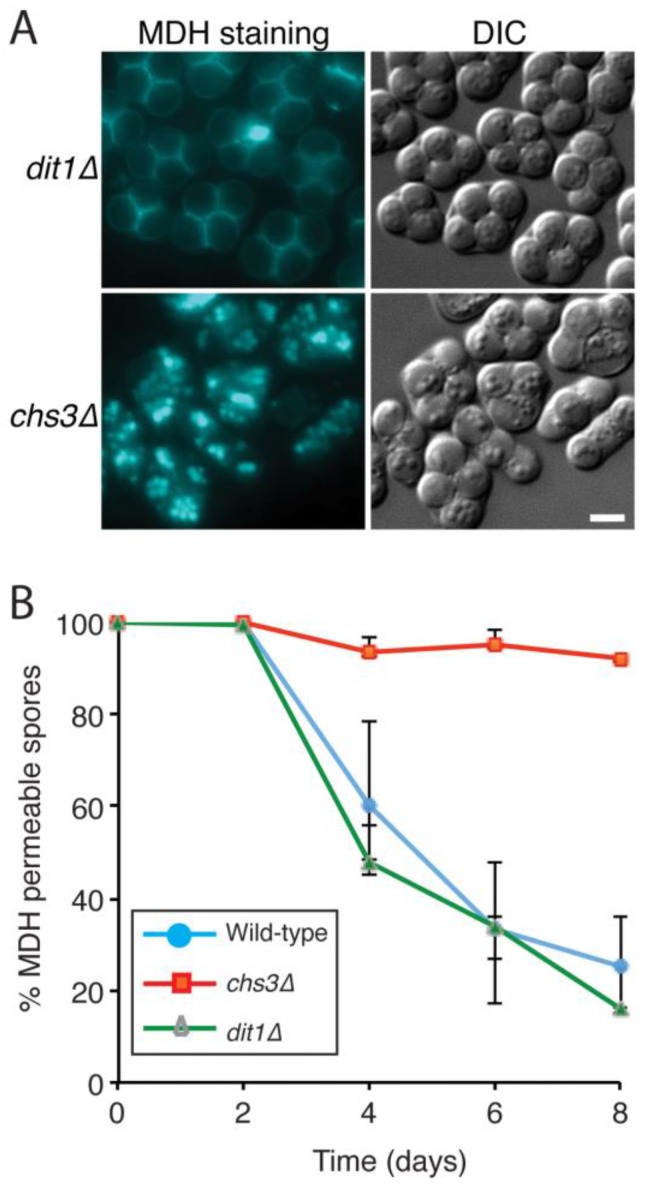
The outer spore wall is required for impermeability to MDH. (**A**) Asci formed by *dit1*∆ (AN264) or *chs3*∆ (AN262) diploids after incubation on SPO plates for eight days were stained with MDH and observed by fluorescence (blue staining) and light microscopy. Scale bar = 3 microns. (**B**) Time course of MDH staining wild-type (AN120), *dit1*∆ (AN264), and *chs3*∆ (AN262) strains were sporulated on SPO plates and at two day intervals, spores were stained with MDH and the percentage of spores showing MDH staining of lipid droplets within the spore was determined. 500 spores were scored at each time point for each strain. Error bars indicate the range of values obtained at each time point over three separate experiments.

If the loss of staining is due to changes in permeability of the spores, the spore wall is likely responsible. A *dit1*∆ mutant strain, lacking the dityrosine layer and a *chs3*∆ mutant strain, which lacks both the chitosan and dityrosine layers [8,29], were examined by MDH staining (Figure 2A). After eight days on SPO medium, the *dit1*∆ spores predominantly lacked MDH staining, similar to the wild-type. By contrast, older spores in *chs3*∆ strain remained as permeable to the dye as newly formed spores. To examine this process more closely, a time course analysis was performed, staining spores of wild-type, *chs3*∆ and *dit1*∆ strains with MDH at intervals over an eight day period (Figure 2B). This analysis revealed that the loss of staining was progressive; over the eight days, the fraction of spores in wild-type displaying intracellular MDH staining diminished from 100% to less than 30%. This change in staining pattern was transient, as spores aged beyond 10 days displayed increasing permeability with time (unpublished obs.). The *dit1*∆ mutant displayed similar kinetics to wild-type cells, with most spores becoming impermeable to the dye after eight days. By contrast, spores in the *chs3*∆ mutant remained almost 100% permeable even after eight days of incubation. These results indicate that some maturation process occurring in the outer spore wall is responsible for impermeability to MDH staining and impermeability to the dye requires the proper assembly of the chitosan layer. However, the dityrosine layer of the spore wall is dispensable for this process.

### 3.2. Acquisition of Impermeability Discriminates between Mutants with Outer Spore Wall Defects

Chitosan is required for the maturation process but dityrosine is not. This could indicate either that chitosan itself is the source of the impermeability or that some non-dityrosine component of the outer wall that is dependent on chitosan for assembly is responsible. A number of mutants have been described that alter the outer spore wall without obviously altering the chitosan layer [14,18]. Genes reported to be important for dityrosine layer formation include *DIT2* and *DTR1* involved in dityrosine synthesis and export, respectively, and *OSW3* and *OSW5*, which were identified on the basis that spore walls in mutants of these genes lack a barrier to the diffusion of protein-sized molecules out of the spore wall, similar to *dit1∆* mutants [12,17,29]. The *osw3∆* mutant, in particular, displays reduced dityrosine fluorescence from the wall. Furthermore, double mutants in the paralogous genes *OSW4 OSW6*, *OSW7 SHE10*, and *NPP1 NPP2*, were all characterized by NMR analysis of their spore walls as lacking dityrosine but retaining both chitosan and Chi [14]. Three additional knockouts of paralogous genes *LDS1 LDS2 RRT8*, *QDR1 QDR3 DTR1*, and *GAT3 GAT4* displayed defects in both dityrosine and Chi [14]. Finally, the *OSW2* gene was identified as a strain that produces spores highly sensitive to ether vapor, suggesting an outer spore wall defect, but displaying no obvious defects or loss of spore wall components [18]. 

**Figure 3:**
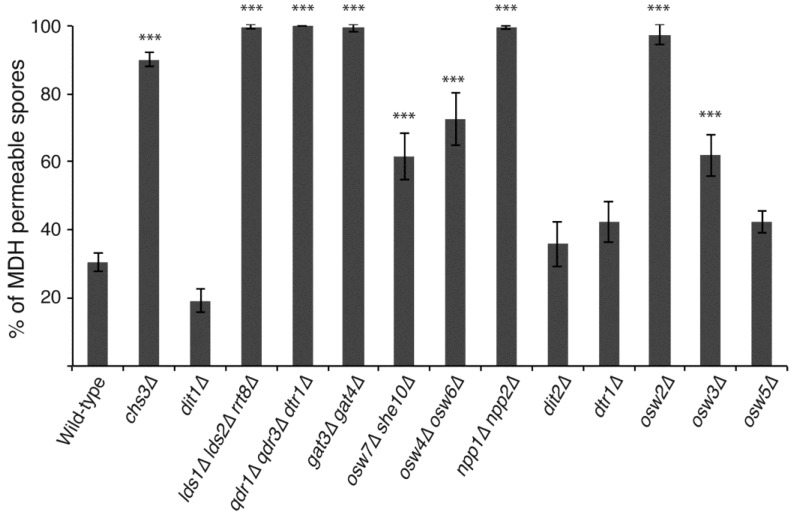
The effects of different outer spore wall mutants on MDH permeability. Wild-type (AN120), *chs3*∆ (AN262), *dit1*∆ (AN264), *lds1*∆ *lds2*∆ *rrt8*∆ (CL6), *qdr1*∆ *qdr3*∆ *dtr1*∆ (CL7), *gat3*∆ *gat4*∆ (CL15), *osw7*∆ *she10* (CL26), *osw4*∆ *osw6*∆ (CL35), *npp1*∆ *npp2*∆ (CL57), *dit2*∆ (ySZ233), *dtr1*∆ (MYA-1810), *osw2*∆ (ySZ265), *osw3*∆ (MYA-1824), and *osw5*∆ (MYA-2022) diploids were incubated on SPO plates for eight days and then tested for MDH staining of lipid droplets within the spores. The percentage of spores displaying internal MDH staining is shown. Values are the averages from four experiments with at least 200 spores scored for each strain in each experiment. Error bars indicate one standard deviation. Asterisks denote strains in which permeability is significantly different from the wild-type (*p* < 0.001 student’s *t*-test).

All of these mutants were examined for MDH staining after eight days of sporulation and the percentage of MDH permeable spores was determined (Figure 3). Of the genes previously implicated as involved in dityrosine layer assembly, spores in strains carrying mutations in *DIT2*, *DTR1*, and *OSW5* all became largely impermeable to MDH, similar to the wild-type and *dit1*∆ strains, consistent with these genes being primarily involved in dityrosine layer formation. The *osw4*∆ *osw6*∆, *osw7*∆ *she10*∆, and *osw3*∆ strains all displayed intermediate levels of permeability between the wild-type and *chs3*∆ controls. This increase in permeability was significantly different from wild-type (*p* < 0.001, student’s *t*-test). The *npp1*∆ *npp2*∆ mutant was as permeable as a *chs3*∆ mutant strain. These results indicate that *OSW3*, *OSW4*/*OSW6*, *OSW7*/*SHE10* and particularly *NPP1*/*NPP2* have effects on the outer spore wall beyond dityrosine assembly. All of the mutants shown to have effects on the presence of Chi in the spore wall (*lds1∆ lds2∆ rrt8*∆, *qdr1∆ qdr3∆ dtr1*∆, *gat3*∆ *gat4*∆) remained completely permeable to MDH. As all of these mutants retain the chitosan layer [14]; this indicates that the presence of chitosan alone is not responsible for the impermeability of the spore wall to MDH and implicates Chi as important for this property of the wall. 

Mutations in *chs3*∆, but not *dit1*∆ are also particularly sensitive to ether vapor [14], paralleling their effects on permeability to MDH. The *osw2∆* mutant, whose primary phenotype is sensitivity to ether vapor, remained permeable to the dye. This further extends the correlation between permeability and ether sensitivity and suggests that *OSW2* is specifically involved in the synthesis of whatever spore wall component is responsible for impermeability to MDH.

### 3.3. De Novo Synthesis of Tyrosine Contributes to Dityrosine Layer Assembly and to Additional Properties of the Spore Wall

**Figure 4:**
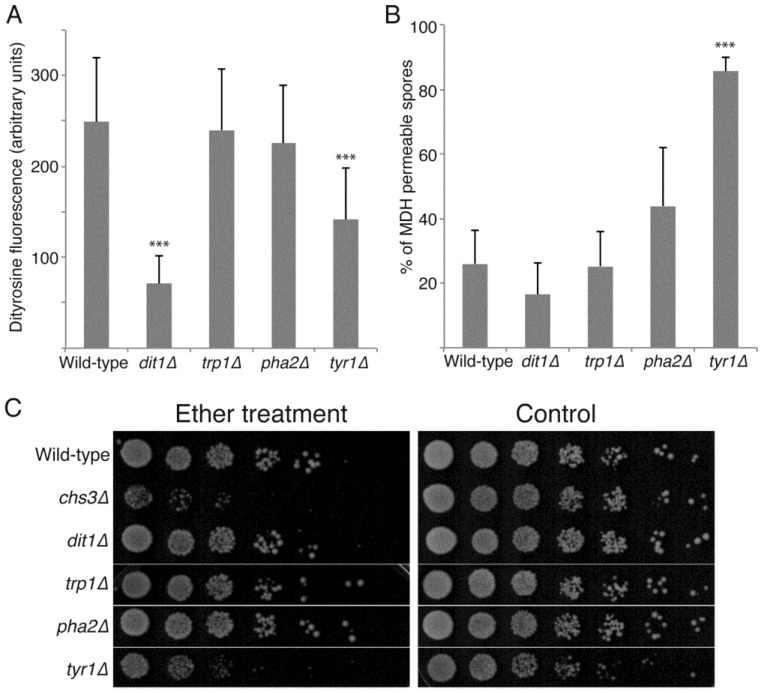
The effect of mutations in aromatic amino acid biosynthesis on outer spore wall formation. (**A**) Wild-type (yKZ107), *dit1*∆ (yZK112), *trp1*∆ (AN120), *pha2*∆ (yKZ109), and *tyr1*∆ (yKZ110) diploids were sporulated for 48 h in liquid culture and the intensity of dityrosine fluorescence in the spore walls was measured by fluorescence microscopy. The average intensity from 20 different spores from each strain is shown. Error bars indicate one standard deviation. Asterisks indicate a significant change from the wild-type (*p* < 0.001 student’s *t*-test). The experiment was performed three times with similar results; one representative experiment is shown. (**B**) The same strains as in (**A**) were examined by MDH staining after eight days incubation on solid SPO medium. The percentage of spores displaying internal MDH staining is shown. Values are the averages from three experiments with 500 spores scored for each strain in each experiment. Error bars indicate one standard deviation. Asterisks denote significant difference from the wild-type (*p* < 0.001 student’s *t*-test). (**C**) Test of ether resistance. The same diploids as in (**A**) plus a *chs3*∆ diploid (yZK111) were sporulated in liquid culture for 48 h. Fivefold serial dilutions of each culture were spotted onto two rich medium (YPD) plates and then one plate (left panel) was exposed to ether vapor for 30 minutes. Both plates were incubated at 30 °C for two days before being photographed.

Metabolomic studies of sporulating yeast cells indicate that, as cells progress through sporulation, there is an influx of carbon into pathways that lead to synthesis of spore wall precursors, both nucleotide sugars and aromatic amino acids [30]. The increased flux into the aromatic amino acid pathway was suggested to indicate synthesis of tyrosine for incorporation into the dityrosine layer of the spore wall. However, aromatic amino acids share common synthesis steps and so these data might also indicate a need for phenylalanine or tryptophan in the synthesis of some spore wall component. To test this directly, strains carrying specific defects in tryptophan (*trp1*∆), phenylalanine (*pha2*∆) or tyrosine (*tyr1*∆) synthesis were sporulated and the spores examined. All three strains efficiently produced visible spores, indicating *de novo* synthesis of these amino acids is not essential for spore formation. Dityrosine fluorescence from the spore walls of each mutant was then quantified by fluorescence microscopy and compared to wild-type and *dit1*∆ strains as controls (Figure 4A). While the *trp1*∆ and *pha2*∆ mutants display levels of dityrosine fluorescence comparable to wild-type, *tyr1*∆ mutants display significantly reduced fluorescence, though not down to the background level seen in *dit1*∆. These results indicate that *de novo* tyrosine synthesis contributes to formation of the dityrosine layer.

The mutants in aromatic amino acid synthesis were further examined for spore wall defects by MDH staining of 8-day old spores and by ether test (Figure 4B,C). The *pha2*∆ and *trp1*∆ spores displayed decreased permeability to MDH similar to wild-type and were resistant to exposure to ether vapor. The *tyr1*∆ mutant, however, displayed increased permeability of the spores and increased ether sensitivity. These phenotypes were stronger than those in the *dit1*∆ mutant, which completely lacks dityrosine. Thus, the use of the MDH staining reveals that the biosynthesis of tyrosine plays an additional role in spore wall assembly beyond simply acting as a precursor for dityrosine synthesis.

## 4. Discussion

Staining with MDH reveals a maturation process in *S. cerevisiae* spore wall development as the spores become progressively impermeable to the dye over time. The basis for this process is unclear and the assay may be somewhat unique to MDH as spores were impermeable to a second lipid droplet dye, BODIPY-TR [14], as soon as they formed (K.Z., unpublished observations). Nonetheless, MDH staining provides a sensitive assay that allows differentiation of the phenotypes of genes affecting outer spore wall assembly.

The fact that *dit1*∆ mutants develop impermeability like wild-type cells indicates that the dityrosine layer is dispensable for this property of the wall. This means that the MDH assay can distinguish whether mutants defective in dityrosine assembly are solely involved in formation of the dityrosine layer or if they have additional roles in wall assembly, suggesting their effects on dityrosine may be more indirect. The latter is certainly the case for mutation of *NPP1/NPP2*. Although earlier biochemical studies had suggested that reduction in dityrosine was the only major change in the outer spore wall in an *npp1*∆ *npp2*∆ strain [14], the permeability of these spores to MDH clearly indicates additional abnormalities in the wall. Similarly, the *osw3*∆, *osw4*∆ *osw6*∆, and *osw7*∆ *she10*∆ mutant strains show intermediate levels of permeability suggesting roles beyond dityrosine assembly. Consistent with this, the *osw4*∆ *osw6*∆ and *osw7*∆ *she10*∆ mutants are more ether sensitive than a *dit1*∆ mutant [14]. Whether the intermediate level of permeability in these mutants indicates that they fail to make some component of the wall or if they have a temporal delay in assembly remains to be determined. Deletions of *OSW5* display somewhat reduced dityrosine fluorescence [17] and impermeability to MDH, which may indicate that *OSW5* is specifically involved in generation of the dityrosine layer. 

In the mutants examined, there is a good correlation between ether resistance and impermeability to MDH staining, though it is not yet clear how these two properties of the outer spore wall are related. The two phenotypes are not perfectly linked as wild-type strains are resistant to ether even when the spores are newly formed and permeable to the dye. It may be that some component of the spore wall confers resistance to ether immediately upon deposition in the wall and then subsequently ‘matures’ to confer impermeability to MDH. There is also a correlation between the absence of Chi and ether sensitivity [14], suggesting the hypothetical component conferring ether resistance and impermeability could be Chi. However, this correspondence is not perfect as mutants like *npp1*∆ *npp2*∆ are ether sensitive and permeable to MDH, but still have Chi present in their spore walls (Figure 3; [14]).

The *OSW2* gene is particularly interesting in regards to the spore wall component responsible for impermeability. Mutants in *OSW2* have no obvious cytological or biochemical defect in the spore wall and yet *osw2*∆ spores are highly ether sensitive and permeable to MDH [18]. This suggests that *OSW2* may play a relatively direct role in promoting these properties of the spore wall. The Osw2 protein is localized to the spore cytoplasm and has homology to ketopantoate reductase and the larger family of NADP dependent dehydrogenase enzymes ([31]; unpublished obs.). Therefore, rather than being directly involved in spore wall assembly, Osw2 could be involved in synthesis of some precursor that is exported from the cytoplasm to the wall similar to dityrosine. Consistent with the idea of export, the *qdr1*∆ *qdr3*∆ *dtr1*∆ strain deficient in multiple transporters [14] displays permeability, suggesting these transporters could be involved in export of the hypothetical Osw2 product.

The phenotypes of the *tyr1*∆ mutant demonstrate that *de novo* synthesis of tyrosine provides material for assembly of the dityrosine layer of the spore wall. Mutants in *TYR1* do not have as severe an effect on dityrosine fluorescence as mutations in *DIT1* indicating that much of the tyrosine for dityrosine synthesis must come from other sources. However, it is noteworthy that *de novo* tyrosine synthesis contributes to the spore wall as sporulation occurs under conditions of nitrogen starvation. This suggests that during sporulation, not only carbon atoms but also scarce nitrogen resources are shunted into the aromatic amino acid biosynthesis pathway to allow for spore wall formation.

In addition to providing tyrosine for dityrosine, the permeability of *tyr1*∆ mutants to MDH indicates additional roles for tyrosine in spore wall synthesis. Given the permeability and ether sensitivity of the *tyr1*∆ mutant, it is tempting to speculate that tyrosine could be a precursor to Chi or some other component made in an *OSW2*-dependent pathway. The ^13^C NMR spectra by which Chi was defined do not include chemical shifts consistent with tyrosine [14], so if tyrosine is a precursor for Chi synthesis, it must be significantly modified. Nonetheless, our results expand the roles of aromatic amino acids in promoting fungal cell wall synthesis.

While spore wall organization is best studied in *S. cerevisiae*; the organization of the wall is likely similar in all ascomycetes. For example, the spore wall of the distantly related fission yeast *Schizosaccharomyces pombe* also has additional layers relative to the vegetative wall and the *S. pombe* spore wall, like the *S. cerevisiae* spore wall, uniquely contains chitosan [32,33,34]. Moreover, the sporulation-induced *mug72* gene of *S. pombe* encodes an enzyme related to *OSW2* [35], raising the possibility that the *OSW2*-generated component of the *S. cerevisiae* spore wall is present in the *S. pombe* wall as well. The outer surface of the *S. pombe* wall is constructed from a protein rather than dityrosine [36]; however, many other ascomycete genomes carry orthologs to the dityrosine synthesis genes *DIT1* and *DIT2* as well as the dityrosine exporter *DTR1* and dityrosine has been detected in several other yeasts [37,38]. Thus, the mechanisms of assembly and properties of the spore wall in *S. cerevisiae* are likely to be a useful model for ascospore walls in other ascomycetes.

## Figures and Tables

**Table 1:** Strains used in this study.

Strain	Genotype	Source
AN120	*MATa/MATα ura3/ura3 leu2/leu2 his3∆SK/his3∆SK trp1::hisG/trp1::hisG ARG4/arg4-NspI lys2/lys2* *ho*Δ*::LYS2/ho*Δ*::LYS2 RME1/rme1*Δ*::LEU2*	[24]
AN117-4B	*MATα ura3 leu2 his3∆SK trp1::hisG arg4-NspI lys2 ho∆::LYS2 rme1*Δ*::LEU2*	[24]
AN117-16D	*MATa ura3 leu2 his3∆SK trp1::hisG lys2 ho*Δ*::LYS2*	[24]
yKZ70	as AN117-16D plus *PET10::PET10-RFP::TRP1*	this study
yKZ72	as AN117-4B plus *PET10::PET10-RFP::TRP1*	this study
yKZ104	as AN120, plus *PET10::PET10-RFP::TRP1*/*PET10::PET10-RFP::TRP1*	this study
AN262	as AN120, plus *chs3*∆::*HIS3MX6*/*chs3*∆::*HIS3MX6*	[25]
AN264	as AN120, plus *dit1*∆::*HIS3MX6*/*dit1*∆::*HIS3MX6*	[25]
CL6	*MATa/MATα HO/HO leu2/leu2 lys2/lys2 URA3::tet0224/URA3::tet0224 lds1*∆*::HIS3MX6/lds1*∆*::HIS3MX6 rrt8*∆*::kanMX6/rrt8*∆*::kanMX6 lds2*∆*::hphMX6/lds2*∆*::hphMX6*	[14]
CL7	*MATa/MATα HO/HO leu2/leu2 lys2/lys2 URA3::tet0224/URA3::tet0224 dtr1*∆*::HIS3MX6/dtr1*∆*::HIS3MX6 qdr3*∆*::kanMX6/qdr*3∆*::kanMX6 qdr1*∆*::hphMX6/qdr1*∆*::hphMX6*	[14]
CL15	*MATa/MATα HO/HO leu2/leu2 trp1/trp1 lys2/lys2 URA3::tet0224/URA3::tet0224 gat4*∆*::HIS3MX6/gat4*∆*::HIS3MX6 gat3*∆*::kanMX6/gat3*∆*::kanMX6*	[14]
CL26	*MATa/MATα HO/HO leu2/leu2 URA3::tet0224/URA3::tet0224 osw7*∆*::HISMX63/osw7*∆*::HIS3MX6 she10*∆*::kanMX6/she10*∆*::kanMX6*	[14]
CL35	as AN120, plus *osw4,6*Δ*::HIS3MX6/osw4,6*Δ*::HIS3MX6*	[14]
CL57	*MATa/MATα HO/HO leu2/leu2 lys2/lys2 URA3:tet0224/URA3:tet0224 npp2*∆*::HIS3MX6/npp2*∆*::HIS3MX6 npp1*∆*::kanMX6/npp1*∆*::kanMX6*	[14]
ySZ233	as AN120, plus *dit2*∆*::kanMX6*/*dit2*∆::*kanMX6*	this study
ySZ265	as AN120, plus *osw2*∆*::hphMX4*/*osw2*∆*hphMX4*	this study
MYA-1810	*MATa/MATa HO/HO his3/his3 trp1/trp1 lys2/lys2 LEU2::P_URA3_-tetR-GFP/LEU2::P_URA3_-tetR-GFP REC8::HA3-URA3/REC8::HA3-URA3 URA3::tetO224/URA3::tetO224 dtr1*∆*::HIS3MX6*/*dtr1*∆*::HIS3MX6*	[26]
MYA-1824	*MATa/MATa HO/HO his3/his3 trp1/trp1 lys2/lys2 LEU2::P_URA3_-tetR-GFP/LEU2::P_URA3_-tetR-GFP REC8::HA3-URA3/REC8::HA3-URA3 URA3::tetO224/URA3::tetO224 osw3*∆*::HIS3MX6*/*osw3*∆*::HIS3MX6*	[26]
MYA-2022	*MATa/MATa HO/HO his3/his3 trp1/trp1 lys2/lys2 LEU2::P_URA3_-tetR-GFP/LEU2::P_URA3_-tetR-GFP REC8::HA3-URA3/REC8::HA3-URA3 URA3::tetO224/URA3::tetO224 osw5*∆*::HIS3MX6*/*osw5*∆*::HIS3MX6*	[26]
yKZ107	as AN120, plus *trp1::hisG::TRP1/trp1::hisG*	this study
yKZ108	as AN120, plus *pha2*∆*::kanMX6*/*pha2*∆*::kanMX6 trp1::hisG::TRP1/trp1::hisG*	this study
yKZ110	as AN120, plus *tyr1*∆*::kanMX6*/*tyr1*∆*::kanMX6 trp1::hisG::TRP1/trp1::hisG*	this study
yZK111	as AN262, plus *trp1::hisG::TRP1/trp1::hisG*	this study
yKZ112	as AN264, plus *trp1::hisG::TRP1/trp1::hisG*	this study
